# Enhanced thermal conductivity of epoxy composites filled with tetrapod-shaped ZnO†

**DOI:** 10.1039/c8ra01470a

**Published:** 2018-03-29

**Authors:** Liangchao Guo, Zhenyu Zhang, Ruiyang Kang, Yapeng Chen, Xiao Hou, Yuming Wu, Mengjie Wang, Bo Wang, Junfeng Cui, Nan Jiang, Cheng-Te Lin, Jinhong Yu

**Affiliations:** Key Laboratory for Precision and Non-Traditional Machining Technology of Ministry of Education, Dalian University of Technology Dalian 116024 China zzy@dlut.edu.cn; Key Laboratory of Marine Materials and Related Technologies, Zhejiang Key Laboratory of Marine Materials and Protective Technologies, Ningbo Institute of Materials Technology and Engineering, Chinese Academy of Sciences Ningbo 315201 China yujinhong@nimte.ac.cn

## Abstract

Epoxy composites with ZnO powders characterized by different structures as inclusion are prepared and their thermal properties are studied. The experimental results demonstrate that the epoxy resins filled by tetrapod-shaped ZnO (T-ZnO) whiskers have the superior thermal transport property in comparison to ZnO micron particles (ZnO MPs). The thermal conductivity of ZnO/epoxy and T-ZnO/epoxy composites in different mass fraction (10, 20, 30, 40, 50 wt%) are respectively investigated and the suitable models are compared to explain the enhancement effect of thermal conductivity. The thermal conductivity of T-ZnO/epoxy composites with 50 wt% filler reaches 4.38 W m^−1^ K^−1^, approximately 1816% enhancement as compared to neat epoxy. In contrast, the same mass fraction of ZnO MPs are incorporated into epoxy matrix showed less improvement on thermal conduction properties. This is because T-ZnO whiskers act as a thermal conductance bridge in the epoxy matrix. In addition, the other thermal properties of T-ZnO/epoxy composites are also improved. Furthermore, the T-ZnO/epoxy composite also presents a much reduced coefficient of thermal expansion (∼28.1 ppm K^−1^) and increased glass transition temperature (215.7 °C). This strategy meets the requirement for the rapid development of advanced electronic packaging.

## Abstract 2

Epoxy composites with ZnO powders characterized by different structures as inclusion are prepared and their thermal properties are studied.

## Graphical abstract

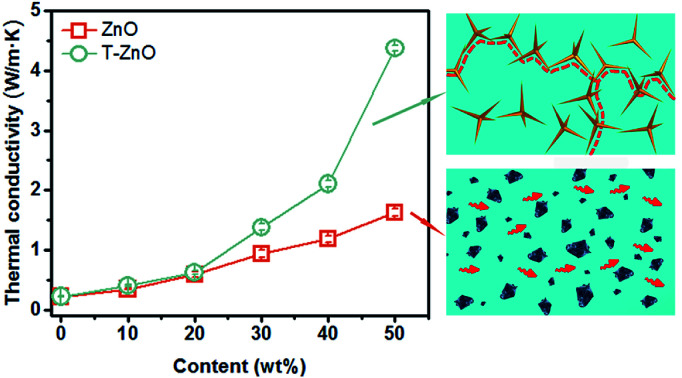

## 1. Introduction

With the development of integrated circuit chips toward higher density and high frequency, more and more waste heat must be removed to make the device work stable. Thus, this has prompted fundamental studies to high thermal conduction in the field of advanced polymeric composites. The polymeric materials have strong potential as thermal management materials because of the ease of their processing, light weight, and low cost.^1^ However, the thermal conductivity of polymers is quite low, *i.e.* in the light of 0.1 W m^−1^ K^−1^ at room temperature, which cannot meet the heat dissipating requirements of modern electronic and electrical systems.^2–4^ Therefore, polymer composites containing ceramic fillers, such as alumina (Al_2_O_3_), aluminum nitride (AlN), boron nitride (BN) and zinc oxide (ZnO), have been the subject of studies to enhance their thermal conductivity.^5–11^ Generally, thermal conductivity of the polymer composites is chosen based on the thermal conductive chains or networks, which are formed between fillers and fillers. However, the quasi-circular ceramic fillers can hardly form thermal conductive chains except if high content fillers is applied.

Tetrapod-shaped ZnO whisker (T-ZnO) is a new type of ZnO whisker with superior performances, such as thermally conductive and other uses.^12–14^ Yuan *et al.*^15^ prepare a phenolic formaldehyde composite with an in-plane thermal conductivity of 1.96 W m^−1^ K^−1^ by compounding with 30 wt% T-ZnO. However, less attention is paid to the formation mechanism of three-dimensional thermal network offered by T-ZnO. Nie *et al.*^16^ investigate and predict the synergistic effect of T-ZnO and BN in the thermal conductive high-density polyethylene composites, but the thermal conductivity of composites with 50 wt% filler is about 1.99 W m^−1^ K^−1^, which is relatively low. In addition, graphene-coated T-ZnO hybrids are fabricated by chemical modification of T-ZnO. Though, a relatively high thermal conductivity of 3.96 W m^−1^ K^−1^ is achieved for the epoxy composite with 55 wt%, while the fabrication process is quite fussy and time consuming.^17^ Based on the previous study, the inclusion of T-ZnO into polymer matrix is still one of the hardest challenges in polymer composites with high thermal conductivity.

In this study, we propose a facile method for the preparation of epoxy composites containing T-ZnO fillers by solution blending process and the fillers are mixed uniformly with epoxy using ethanol as the solvent. A high thermal conductivity of 4.38 W m^−1^ K^−1^ is achieved for the epoxy composite with 50 wt% T-ZnO filler, which is higher than previous references at the same content. The interconnected network of T-ZnO fillers in epoxy matrix results in not only enhancing thermal transportation properties but also retaining low coefficient of thermal expansion (CTE) performance. The characteristics of high thermal conductivity and low CTE of T-ZnO/epoxy composites make it more easily applicable in thermal management of power devices and other electrical devices.

## 2. Experimental

### 2.1 Materials

The ZnO powders with an average diameter of 5 μm were obtained from Forsman Scientific (Beijing) Co., Ltd. China. Tetrapod-shaped ZnO (T-ZnO) whiskers were provided from by Chengdu Crystrealm Co., Ltd. China. A cycloaliphatic epoxy resin (6105, DOW Chemicals, USA) along with hardener of methyl-hexahydrophthalic anhydride (MHHPA, Shanghai Li Yi Science & Technology Development Co. Ltd., China) was used in the present study. Neodymium(iii) acetylacetonate trihydrate (Nd(iii)acac) purchased from Aldrich Chemicals was used as latent catalyst.

### 2.2 Preparation of epoxy composites

**Fig. 1:**
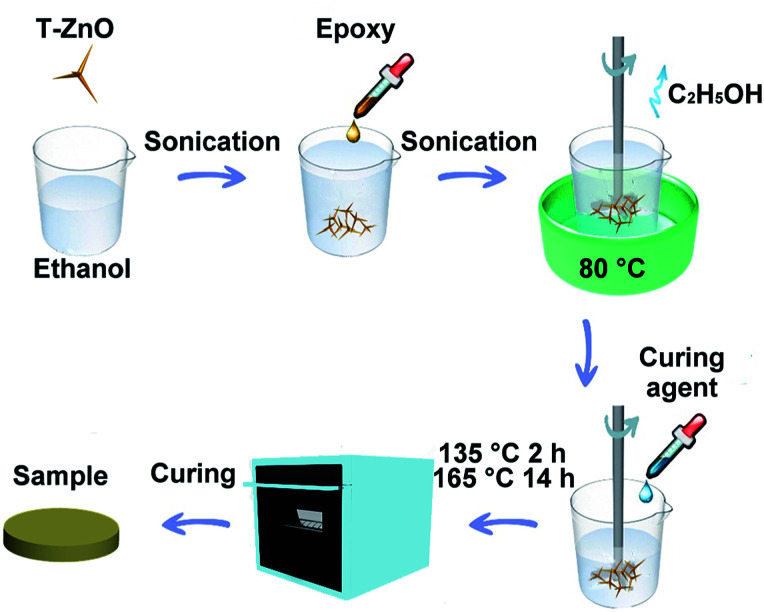
Schematic illustration of the preparation process of T-ZnO/epoxy composites.

The epoxy composites with different T-ZnO loadings were prepared as follows. Firstly, the required ratio of Nd(iii)acac (0.1%) was added to the epoxy resin, stirred and degassed at 80 °C in a three-necked flask. The homogeneous solution was then cooled down to ambient temperature. Secondly, a desired amount of T-ZnO (10, 20, 30, 40, and 50 wt%) was ultrasonically dispersed in ethanol for 1 h at room temperature and then added into the predetermined amount of epoxy resin (200 g). The obtained mixture was then placed in a beaker with vigorous mechanical stirring at 80 °C in water bath until complete evaporation of ethanol. Curing agent (MHHPA) was added at a ratio of 100 : 95 (epoxy : curing agent) by weight into the beaker and was stirred for 20 min. It was further degassed in the vacuum oven for 10 min to remove air bubbles. Finally, the mixture was poured onto preheated stainless steel molds, pre-cured in an oven at 135 °C for 2 h, followed by post-cure at 165 °C for 14 h. The molds were left in the oven and allowed to cool gradually to room temperature and then polished with emery paper for different characterizations. For comparison, the epoxy composites with ZnO were also fabricated with the same procedures as described above. For the sake of convenience, the composites containing ZnO and T-ZnO were denoted as ZnO/epoxy composites and T-ZnO/epoxy composites, respectively. The experiment details of the process of epoxy composites are shown in Fig. 1.

### 2.3 Characterizations

The morphology of the ZnO, T-ZnO and the fractured surface of composites were examined by field emission scanning electron microscopy (FE-SEM, QUANTA FEG250, USA) equipped with energy dispersive spectroscopy (EDS) at an acceleration voltage of 20 kV. Samples that were broken and the fractured surface was coated with a thin gold layer to avoid accumulation of charge. X-ray photoelectron spectroscopy (XPS) was carried out with a Kratos AXIS Ultra DLD spectrometer, using AlKα excitation radiation (*hν*: 1253.6 eV). The X-ray diffraction (XRD) patterns of the samples were recorded on a D8 DISCOVER with GADDS (BRUKER Ltd. Germany) with CuKα radiation (*λ* = 1.5406 Å). The scanning was performed from 10° to 80° with a speed of 4° min^−1^ at room temperature. Thermal conductivities of the composites were measured using the light flash apparatus LFA 467 NanoFlash® (NETZSCH, Germany). The IR-photos were captured by infrared camera (Fluke, Ti400, USA). Differential scanning calorimetry was performed by a Pyris Diamond DSC (Perkin-Elmer, USA) from 20 to 250 °C at a heating rate of 10 °C min^−1^ under a nitrogen atmosphere to study glass transition temperature (*T*_g_) of the composites. The CTE measurements were performed on a thermal mechanical analyzer (TMA 402F1/F3, NETZSCH, Germany) from 310 to 460 K at a heating rate of 5 K min^−1^. Thermogravimetric analysis (TGA) was carried out using TG 209 F3 thermo-analyzer (NETZSCH, Germany). The temperature range was from 50 to 800 °C at a ramp rate of 10 °C min^−1^ in nitrogen atmosphere. Dynamic mechanical analysis (DMA) were performed on a DMA Q800 dynamic mechanical analyzer (TA instruments, USA) operating in the tension mode at an oscillation frequency at 1 Hz.

## 3. Results and discussion

### 3.1 Characterization of ZnO filler

**Fig. 2:**
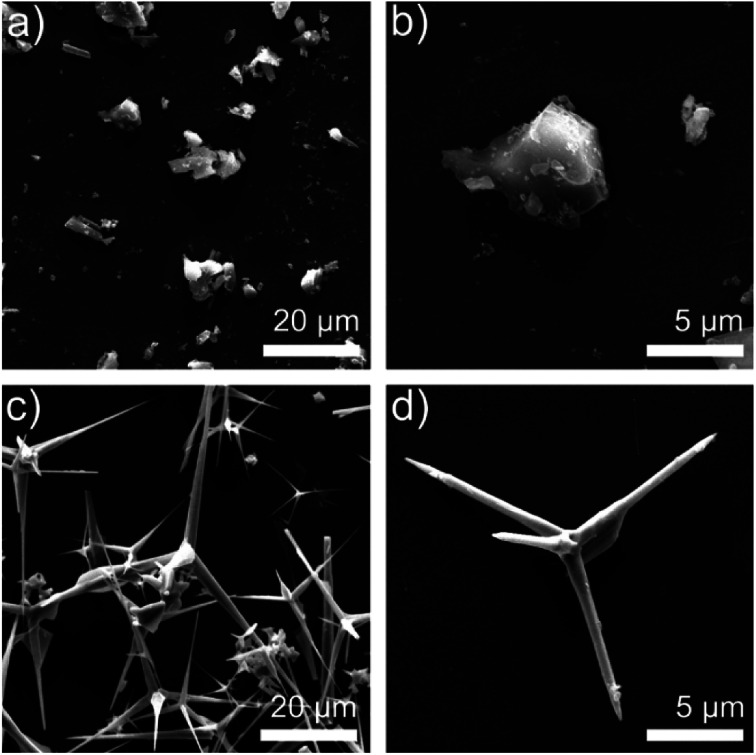
(a) SEM images of ZnO MPs (b) SEM images of single ZnO MP; (c) SEM images of T-ZnO (d) SEM images of single T-ZnO.

The different structure of ZnO MPs and T-ZnO can be observed in Fig. 2. Fig. 2a and b shows the SEM image of ZnO MPs dispersed in ethanol using sonication–centrifugation process. The high-resolution SEM image of ZnO MPs indicates that the particles (size 2–10 μm) have irregular particle size and can be easily aggregated. The range sizes of particles are marked and calculated by one after another, and are taken the average on the basis of the longest axis and the most narrow axis. The aspect ratio of ZnO MPs is difficult to determine, but it can be estimated that the aspect ratio of ZnO MPs is approximately from 1.5 to 1.7. In Fig. 2c and d, T-ZnO is composed of the needle (size 2–10 μm) and root (diameter 0.2–1 μm). The aspect ratio of T-ZnO is calculated about 10, which is considered beneficial to form the thermal conductive networks when filled in epoxy in theory.^18^ Also, the XRD pattern of ZnO MPs exhibits characteristic peaks at 31.9° (100), 34.6° (002), 36.4° (101), 47.6° (102) and 56.7° (110) in Fig. S1a.†^19^ The T-ZnO presents a XRD pattern and Raman spectroscopy similar to that of ZnO MPs, as shown in Fig. S1a and b,† which is an evidence of the same crystal form compared with ZnO MPs. The Raman spectra is an effective and clipping method tool to investigate the crystalline of ceramics.^20,21^ T-ZnO exhibits sharp peak at 437.9 cm^−1^, corresponding to its nonpolar optical phonon E_2_ (high) mode.^22^ Fig. S1c and d† show the dispersion states of the ZnO MPs and T-ZnO in ethanol after standing for 10 min and 12 h.

### 3.2 Morphology of epoxy composites

**Fig. 3:**
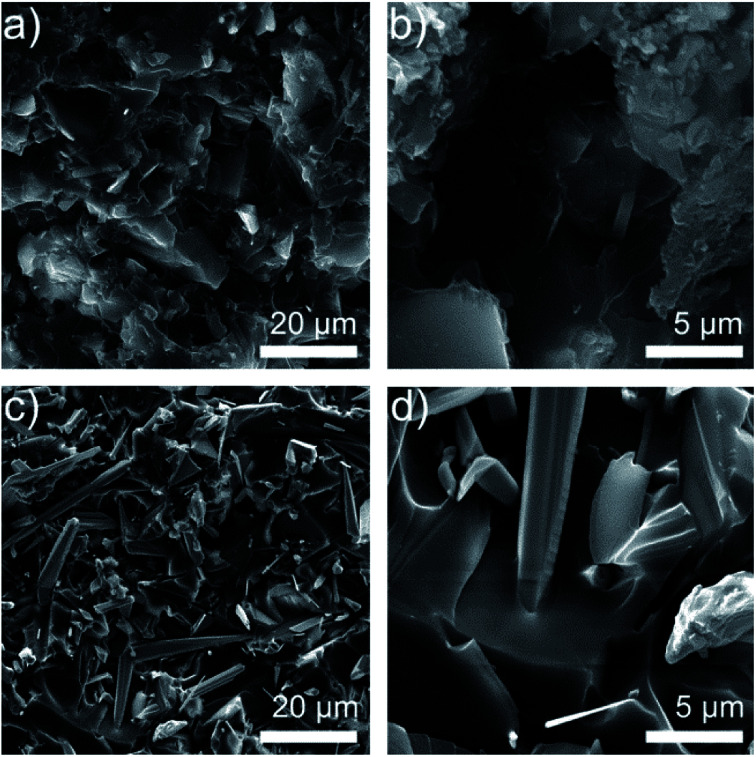
(a) Low resolution SEM images of 50% ZnO/epoxy composites (b) high resolution porous structure in ZnO/epoxy composites (c) low resolution SEM images of 50% T-ZnO/epoxy composites (d) high resolution SEM images of T-ZnO can perfect be wrapped with epoxy.

Fig. 3 reveals SEM images of fracture surfaces of the ZnO/epoxy and T-ZnO/epoxy composites with 50 wt% fillers in order to demonstrate the distribution and structure of fillers in epoxy composites. Fig. 3a and b show that there are some cracks and porous structure on the regions of the fracture surface in ZnO/epoxy composites, indicating a week interfacial interaction between epoxy matrix and ZnO MPs. Unlike epoxy composites filled with ZnO MPs, it can be clearly observed that the pattern of cross section has a smooth surface with the uniform dispersion of T-ZnO and T-ZnO can perfectly be wrapped with epoxy without crack in Fig. 3c and d. It is suggested that the needle of T-ZnO formed a three-dimensional network and a strong interfacial interaction between matrix and surface of T-ZnO in Fig. 3d, which can be concluded as the critical factor for thermal properties. Meanwhile, XPS analysis of T-ZnO/epoxy and ZnO/epoxy composites are employed to further verify the surface composition and investigate the functional groups and C_1s_ high-resolution results are shown in Fig. S2.† The spectra of C_1s_ composes of three bonds centered at 284.6, 285.8, and 287.8 eV, which corresponds to C–C, C–O, C=O, respectively.

**Fig. 4:**
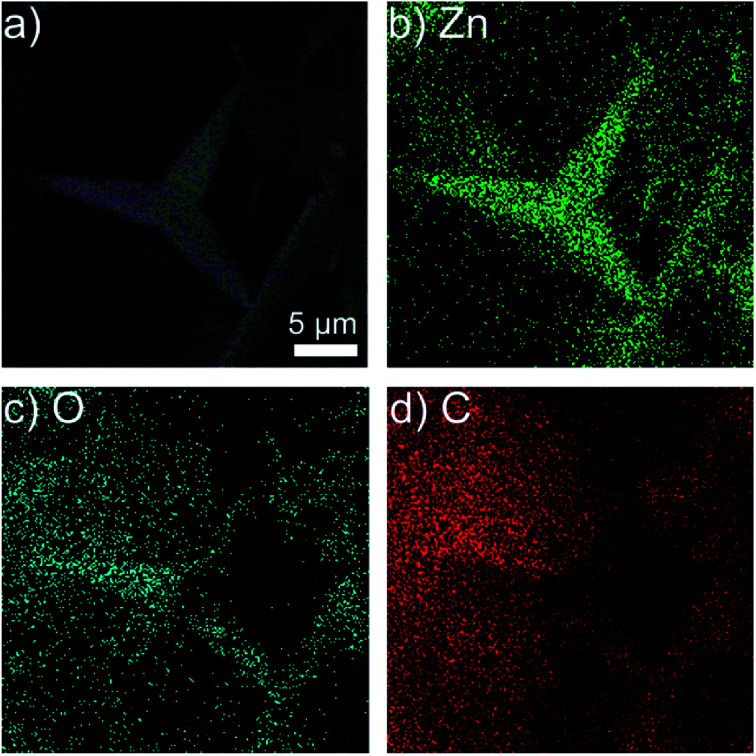
EDS images of (a) 50% T-ZnO/epoxy composites; the distribution of (b) Zn element; (c) O element and (d) C element.

The energy dispersive spectrometer (EDS) of T-ZnO/epoxy observes that there are only Zn, O, C elements in the composites in Fig. 4. Compared with ZnO/epoxy composites in Fig. S3,† the content of Zn, O, C in T-ZnO/epoxy composites are approximately equal with those in ZnO/epoxy. Zn elements in T-ZnO/epoxy composites are continuous in the composites which display the outline of a T-ZnO particle. If the quantity of T-ZnO is further increased and reaches the percolation threshold, local chain and network would mutually bridge to generate the whole network. While O and C elements are randomly distributed in the matrix which is similar to ZnO/epoxy composites. It is means that ZnO MPs are analogous to the “sea-island” structure.

### 3.3 Thermal properties of epoxy composites

**Fig. 5:**
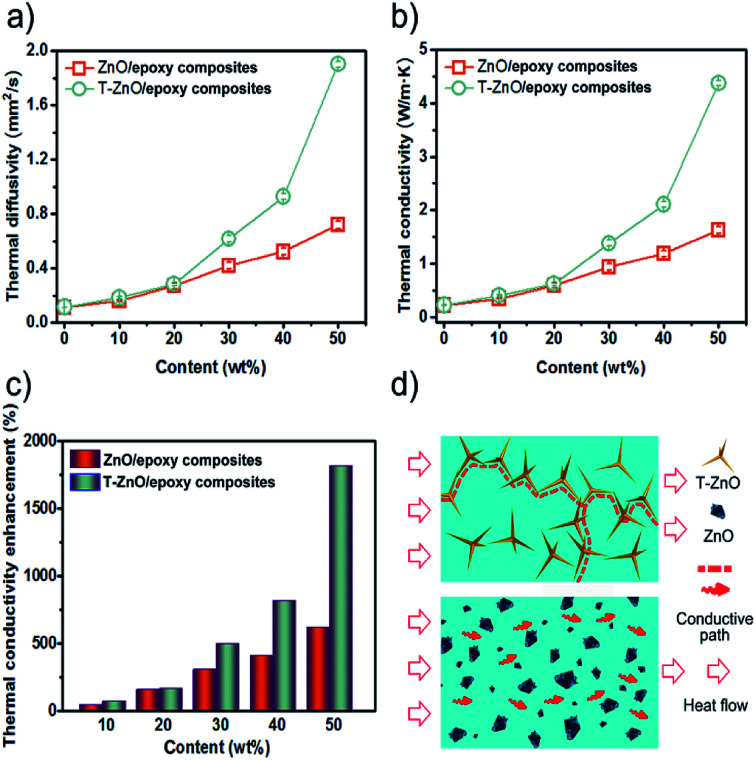
(a) Thermal diffusivity and (b) thermal conductivity of epoxy composites as a function of ZnO MPs or T-ZnO content; (c) thermal conductivity enhancement (TCE) of epoxy composites compared to neat epoxy; (d) the model of heat flow for the epoxy composites.

In order to enhance the thermal conductivity, not only the thermal network maximized through high filler loading, but also the uniform dispersion in polymer matrix is the key factor.^23^ We adopted a transient laser method to indirectly estimate the thermal conductivity of the neat epoxy and its composites. Fig. 5a and b present thermal diffusivity and thermal conductivity of neat epoxy and epoxy composites with 10–50 wt% filler loading. As can be shown, both the thermal diffusivity and conductivity of the samples shows extraordinary increase with increase ZnO and T-ZnO content. The thermal diffusivity of T-ZnO/epoxy composite increased from 0.11 mm^2^ s^−1^ (neat epoxy) to 0.18 mm^2^ s^−1^ (74.6% enhancement) with the addition of 10 wt% T-ZnO. As the content of T-ZnO is further increased to 50 wt%, the thermal diffusivity is improved from 0.11 mm^2^ s^−1^ to 1.91 mm^2^ s^−1^, and the thermal conductivity was improved from 0.22 W m^−1^ K^−1^ to 4.38 W m^−1^ K^−1^. At 50 wt% T-ZnO loading, the thermal conductivity of T-ZnO composite is enhanced significantly by 1817% compared to that of neat epoxy, as illustrated in Fig. 5c. Moreover, a sharply increase on thermal diffusivity and thermal conductivity is observed when the loading fraction of T-ZnO increased from 30 to 50 wt%. It is suggested that the percolation threshold is obtained at ∼30 wt% and T-ZnO three-dimensional network is formed. For comparison, the thermal conductivity of ZnO/epoxy composites was also determined with the same procedure. The value is 1.623 W m^−1^ K^−1^ at 50 wt%, lower than that of T-ZnO/epoxy composites at the same fraction, only 624% comparing to that neat epoxy, as shown in Fig. 5b and c. Moreover, compared to ZnO/epoxy composites, T-ZnO/epoxy composites achieve higher thermal conductivity, which is because of several reasons as following: (a) the aspect ratio of T-ZnO is about 5 times more than ZnO MPs; (b) the heat conductive path and three-dimensional network between T-ZnO and T-ZnO and (c) a better interaction between T-ZnO with the epoxy matrix.^24^

In order to further understand the thermal conductive mechanism of epoxy composites with regard to phonon, it is believed that phonon plays a significant role in heat conduction of majority polymer composites. Meanwhile, both the harmonic or anharmonic phonon–phonon interaction at high temperatures and the scattering of the phonon by the crystal boundaries at low temperatures determine the thermal conductivity of semiconductors.^25^ Neat epoxy is not a good heat conductor due to the low crystallinity and phonon scattering of the randomly entangled molecule chains.^26^ Introducing semiconductors of high thermal conductivity, such as ZnO (∼60 W m^−1^ K^−1^)^27^ into epoxy can solve this problem. The results show that the low loading T-ZnO in the polymer matrix cannot contact with each other just like “islands” in the sea. If the quantity of T-ZnO is sharply increased and reaches the percolation threshold, T-ZnO would built efficient thermal conductive network and greatly enhanced thermal conductivity of the matrix.^28^ In addition, the densely packed structure formed upon vacuum filtration decreased the interfacial thermal resistance, which is another reason for the high thermal conductivity. In order to visualized representation the superiority of ZnO MPs as compared with T-ZnO, the thermal conduction model of ZnO/epoxy and T-ZnO/epoxy composites was proposed to present the thermal path of epoxy composites as shown in Fig. 5d. Also, it can demonstrate that the ZnO MPs cannot contact with each other and the polymer layers stop the ZnO MPs from forming efficient thermal conduction pathway in epoxy, even though the loading is 50 wt%. Comparison with T-ZnO/epoxy composites, that is the reason ZnO/epoxy composites have relatively lower thermal conductivity in arbitrarily loadings. Moreover, the phonon mismatch between ZnO MPs and epoxy matrix leads to a large thermal interface resistance. Phonon mismatch shows that phonon can hardly be absorbed by crystalline structure in the procedure of transmission, which means, the energy phonon carries is not sensitively to the crystal lattice, which is in good agreement with the aforementioned SEM results (as shown in Fig. 3).^28^

**Fig. 6:**
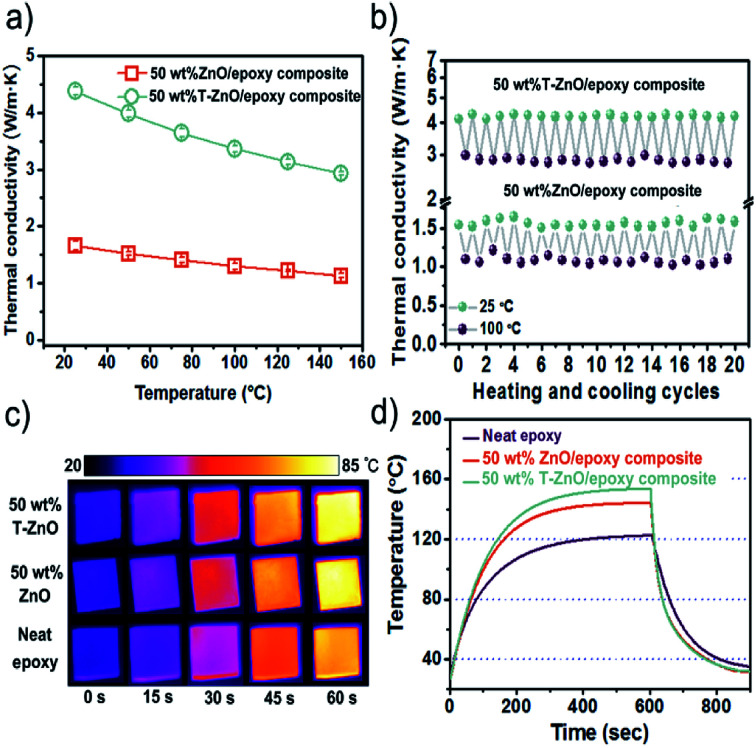
(a) Thermal conductivity of epoxy composites as a function of test temperature; the thermal stability of epoxy composites: (b) thermal conductivity of heating/cooling cycles alternating between 25 °C and 100 °C; (c) infrared images, (d) surface temperature variation with time upon heating and cooling event.

As shown in Fig. 6a, the effect of temperature on thermal conductivity is investigated at 50 wt% loading of T-ZnO and ZnO MPs. It is suggested that the thermal conductivity of composites is in the order of ZnO/epoxy < T-ZnO/epoxy under the same temperature. For the T-ZnO/epoxy composites, thermal conductivity is confirmed to be 4.38 W m^−1^ K^−1^ at 25 °C and decreased with temperature over the temperature range investigated. Similarly, the thermal conductivity of ZnO/epoxy composites shows the same trend with the increase of temperature. It has been demonstrated that crystalline materials exhibit a decrease in thermal conductivity with increasing temperature because of Umklapp phonon scattering, while the thermal conductivity of the amorphous materials increases with increasing temperature.^29^Fig. 6b shows the thermal conductivity changes only slightly change after 20 heating/cooling cycles alternating between 25 °C and 100 °C, suggesting good thermal stability for epoxy composites in this temperature range.

An infrared camera was used to record the temperature response difference during heating, as shown in Fig. 6c. There were three samples vertically placed on the same heater in order of 50 wt% T-ZnO/epoxy composites, 50 wt% ZnO/epoxy composites and neat epoxy from top to down. Infrared camera images showing the color change from blue to red by 60 s which represents that the temperature of the heater increased from room temperature (25 °C) to 100 °C. It is implied that the surface of the sample become more hot with increasing heating time. Moreover, the color of epoxy composites is much brighter comparing to neat epoxy. After 60 s, the surface of T-ZnO/epoxy is 84.4 °C and that of ZnO/epoxy is 81.9 °C, compared that neat epoxy shows a slight orange color and its surface temperature is 74.6 °C. The results give us an interpretation that the better heat dissipation ability of epoxy composites than neat epoxy, especially for the T-ZnO/epoxy composites, which is in accordance with the thermal conductivity values shown in Fig. 5b. In order to verify the effect on heat dissipation, the system with a hot plate, a thermocouple element and versatile voltmeter are employed to get quantitative results of heating and cooling process. As shown in Fig. 6d, neat epoxy rises to 122.4 °C by the end of 601 s, which is consistent with infrared images. While it can be observed that the highest temperature of T-ZnO/epoxy composites is 153.6 °C, compared to that of ZnO/epoxy is 144.3 °C. After 601 s heating, the samples are cooled down to a room temperature plate in several seconds. The cooling data with differential treatment ranging from 601 to 750 s are shown in Fig. 6d. Obviously, we found that T-ZnO/epoxy composites is the fastest one cooling down to room temperature, followed by ZnO/epoxy, and neat epoxy. In brief, epoxy composites show better heat dissipating behaviors comparing with neat epoxy. Among all of composites, T-ZnO/epoxy composites are illuminated to be the most excellent, which indicates that T-ZnO are the most promising filler to enhance the thermal propagation of epoxy composite.

**Fig. 7:**
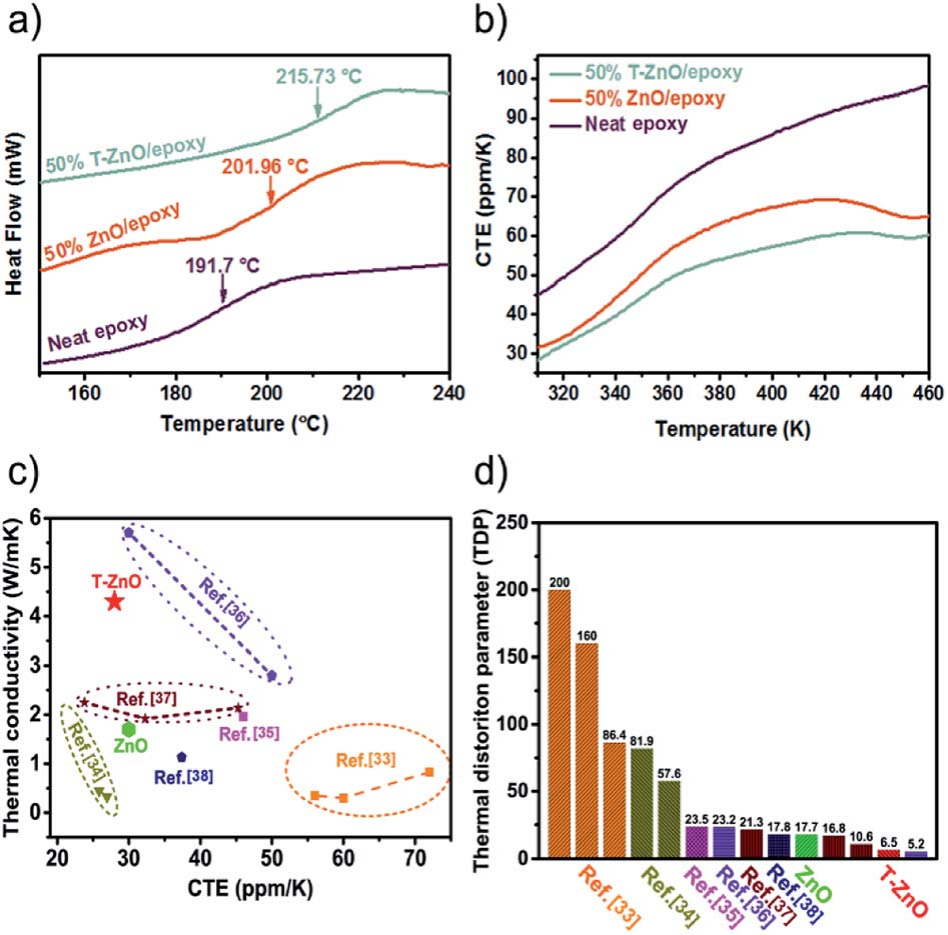
Neat epoxy and epoxy composites: (a) DSC curves, (b) CTE curves; comparison of (c) thermal conductivity *versus* CTE and (d) TDP of the T-ZnO/epoxy and ZnO/epoxy composites with various engineering materials.

Fig. 7a shows the DSC curve of the neat epoxy, ZnO/epoxy and T-ZnO/epoxy at 50 wt% loading, respectively. The glass transition temperatures (*T*_g_) appear over a wide temperature range, because the level of cross-linking in the epoxy matrix is not accordant. It is well-known that the *T*_g_ of epoxy and its composites is bound up with the cross-linking density.^30^ The *T*_g_ of the samples was investigated in temperature range 150–250 °C base on DSC curves. It is observed that the *T*_g_ of neat epoxy is 191.7 °C. While the *T*_g_ of the T-ZnO/epoxy and ZnO/epoxy is 215.7 °C and 201.9 °C, respectively. The *T*_g_ of the ZnO/epoxy and T-ZnO/epoxy composite is increased by 10.2 °C and 24.0 °C, respectively in compared with neat epoxy. The glass transition temperatures is shifted to higher temperature with the addition of T-ZnO and ZnO MPs into the epoxy matrix indicating that a strong interface by reacting with matrix molecules during curing process. This creates more barriers to restrict the motion of macromolecular chain, leading to the higher *T*_g_ and promoting thermal stability. The positive effect can also be found from TGA and DMA curves of epoxy composites, as shown in Fig. S4 and S5.† Fig. S5a† shows storage modulus of neat epoxy and its composites at 50 wt%. Meanwhile, the results show that storage modulus of neat epoxy and composites is in the order of neat epoxy < ZnO/epoxy < T-ZnO/epoxy under the same temperature. Two reasons may account for the enhancement in the storage modulus. First, the applied stresses are expected to be easily transferred from the matrix onto the hybrid fillers due to the high surface area of hybrid fillers. Second, the strong covalent bonding formation between hybrid fillers and epoxy matrix reduced the mobility of the local matrix during the process of epoxy curing reaction.^31,32^ The tan delta of epoxy composites in Fig. S5b† is also consistent with the glass transition temperatures.

In addition, the coefficient of thermal expansion (CTE) of polymer composites is important for electronic packaging. Fig. 7b shows CTE results of neat epoxy and epoxy composites with 50 wt% filler. All the curves show a slowly increase after the temperature upgrades from 360 K. In addition, the T-ZnO/epoxy composite shows lower slope and maintains the slope up to 460 K, which is lower than that of ZnO/epoxy composite in any temperature. Packaging materials with lower CTE value is expected to have lower thermal strain, which will find potential application as heating sinks structural material with improved thermal properties. Furthermore, compared with other particles in epoxy composites, we noticed that the CTE of our samples are much lower as shown in Fig. 7c. The CTE of T-ZnO/epoxy composites at 50 wt% loading and room temperature is 27.9 ppm K^−1^, compared to conventional epoxy based composites, which is the lower CTE value.^33–38^ Also, this outstanding performance combining with high thermal conductivity result in lower TDP, which is a prominent indicator at the engineering-scale and be defined as following eqn (1)1
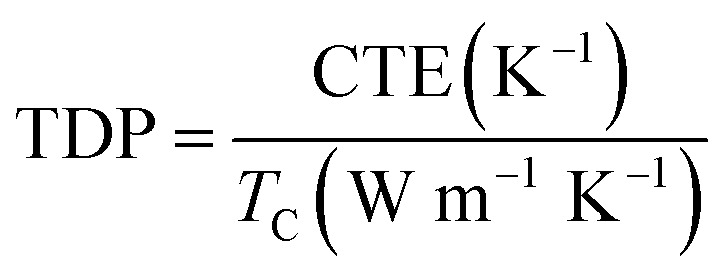


TDP is a prominent indicator of the temperature-induced distortion in a material in applications such as metrology, optics and high-precision engineering.^39^ As shown in Fig. 7d, the lower TDP of composite predicts greater thermal stability, on account of the high thermal conductivity and low CTE of the T-ZnO/epoxy composite, because the efficient conductance pathway formed by the T-ZnO play an important role in determining the thermal stability.^40^ Polymer composites with high thermal conductivity and low TDP would have application prospects in the field of LED, CPU and so on.

## 4. Conclusions

In summary, a facile method to prepare epoxy composites has been demonstrated. The epoxy composite filled by T-ZnO fillers has the superior thermal property in comparison to ZnO MPs. The thermal conductivity of T-ZnO/epoxy with 50 wt% filler was 4.38 W m^−1^ K^−1^, which is enhanced 1817% compared to that of neat epoxy. It is found that T-ZnO/epoxy composite retains the low coefficient of thermal expansion. This strategy meets the requirement for the rapid development of advanced electronic packaging.

## 5. Author contributions

Zhenyu Zhang and Jinhong Yu supervised the projects. Liangchao Guo and Ruiyang Kang carried out the experiments. Jinhong Yu, Liangchao Guo, Ruiyang Kang, Yapeng Chen, Xiao Hou, Yuming Wu, Mengjie Wang, Bo Wang, Junfeng Cui, Nan Jiang and Cheng-Te Lin analyzed the mechanism and thermal properties. All authors discussed the results and commented on the manuscript.

## Conflicts of interest

There are no conflicts to declare.

## Supplementary Material

RA-008-C8RA01470A-s001
