# Supplementary material for: Enhanced thermal conductivity of epoxy composites filled with tetrapod-shaped ZnO
Source: RSC Adv. 2018 Mar 29;8(22):12337–43. doi: 10.1039/c8ra01470a (PMC9079263; doi:10.1039/c8ra01470a)
Supplement: RA-008-C8RA01470A-s001 [file RA-008-C8RA01470A-s001.pdf]

Electronic Supplementary Material (ESI) for RSC Advances.
This journal is © The Royal Society of Chemistry 2018

SUPPORTING INFORMATION

Enhanced Thermal Conductivity of Epoxy Composites Filled with Tetrapod-shaped ZnO

Liangchao Guo [a,b], Zhenyu Zhang [a,*], Ruiyang Kang [a,b], Yapeng Chen [b], Xiao Hou [b], Yuming Wu [b], Mengjie Wang [b], Bo Wang [a,b], Junfeng Cui [a,b], Nan Jiang [b], Cheng-Te Lin [b], Jinhong Yu [b,*]

[a] Key Laboratory for Precision and Non-Traditional Machining Technology of Ministry of Education, Dalian University of Technology, Dalian 116024, China

[b] Key Laboratory of Marine Materials and Related Technologies, Zhejiang Key Laboratory of Marine Materials and Protective Technologies, Ningbo Institute of Materials Technology and Engineering, Chinese Academy of Sciences, Ningbo 315201, China

Corresponding author, Electronic mail: zzy@dlut.edu.cn; yujinhong@nimte.ac.cn.

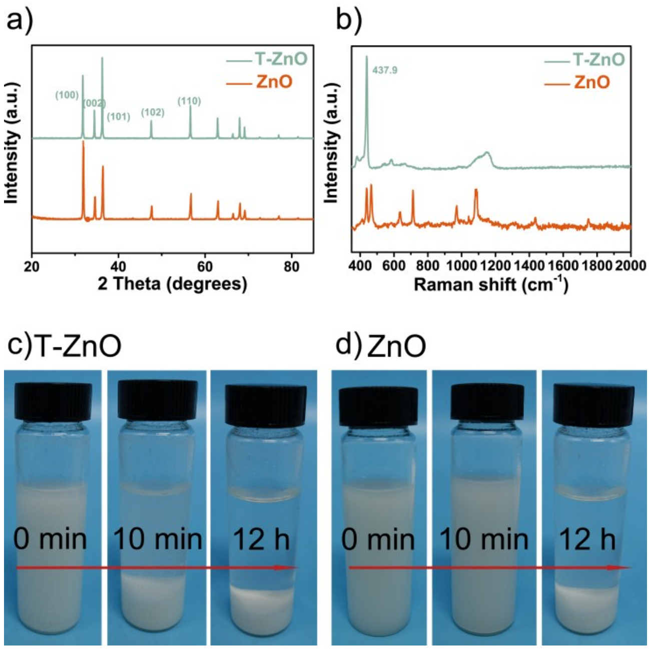

Figure S1. (a) The XRD pattern and (b) the Raman spectra of ZnO and T-ZnO; the dispersion states in ethanol after 10 min and 12 h of (c) T-ZnO and (d) ZnO.

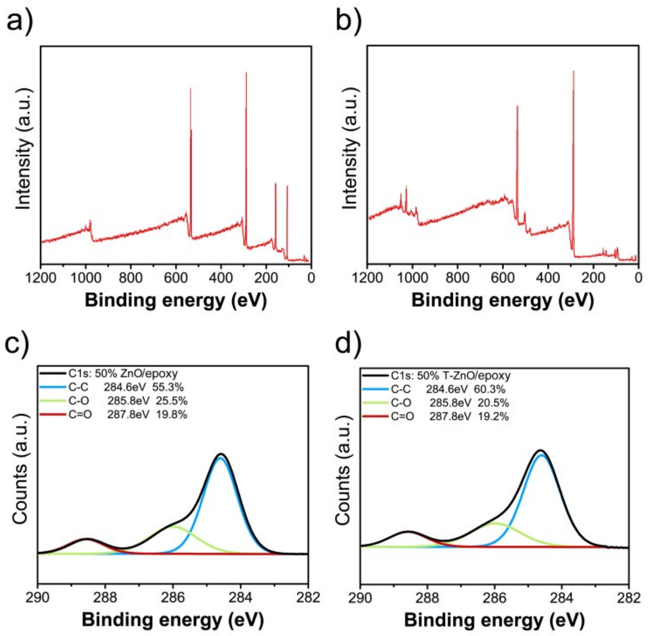

Figure S2. (a) and (b) XPS survey scans of ZnO/epoxy and T-ZnO/epoxy composites; (c) and (d) the corresponding C1s XPS spectra of ZnO/epoxy and T-ZnO/epoxy composites.

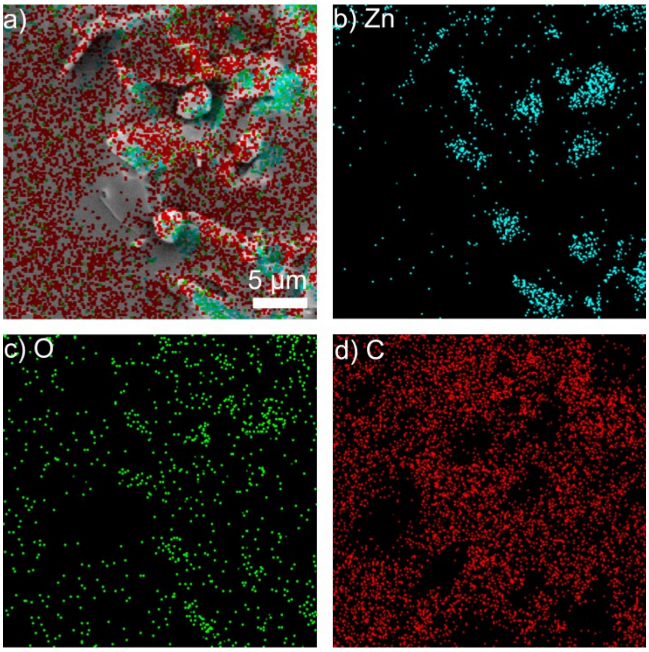

Figure S3. EDS images of (a) 50 wt% ZnO/epoxy composites; the distribution of (b) Zn element; (c) O element and (d) C element.

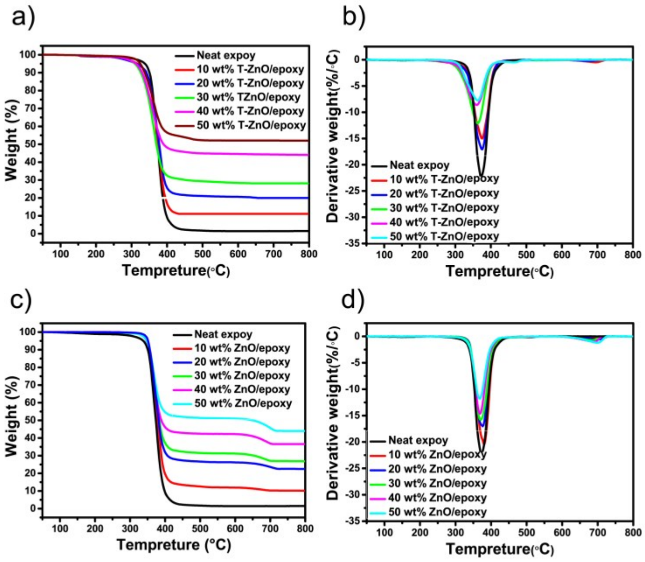

Figure S4. (a) TGA and (b) DTG curves of neat epoxy and T-ZnO/epoxy composites; (c) TGA and (d) DTG curves of neat epoxy and ZnO/epoxy composites.

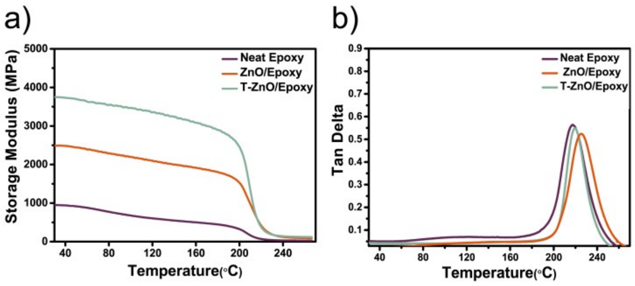

Figure S5. (a) Storage modulus and (b) loss factors of neat epoxy and its composites with 50 wt% fillers.
